# Seroprevalence of antibodies to enterovirus 71 and coxsackievirus A16 among people of various age groups in a northeast province of Thailand

**DOI:** 10.1186/s12985-018-1074-8

**Published:** 2018-10-16

**Authors:** Hatairat Lerdsamran, Jarunee Prasertsopon, Anek Mungaomklang, Chompunuch Klinmalai, Pirom Noisumdaeng, Kantima Sangsiriwut, Boonrat Tassaneetrithep, Ratigorn Guntapong, Sopon Iamsirithaworn, Pilaipan Puthavathana

**Affiliations:** 1 Center for Research and Innovation, Faculty of Medical Technology, Mahidol University, Nakhon Pathom, 73170 Thailand; 2 Debaratana Nakhon Ratchasima Hospital, Nakhon Ratchasima, 30280 Thailand; 3 Department of Pediatrics, Faculty of Medicine, Ramathibodi Hospital, Mahidol University, Bangkok, 10400 Thailand; 4 Faculty of Public Health, Thammasat University (Rangsit Center), Khlong Luang, Pathum Thani, 12121 Thailand; 5 Department of Preventive and Social Medicine, Faculty of Medicine Siriraj Hospital, Mahidol University, Bangkok, 10700 Thailand; 6 Center of Research Excellence in Immunoregulation, Faculty of Medicine Siriraj Hospital, Mahidol University, Bangkok, 10700 Thailand; 7 National Institute of Health, Department of Medical Sciences, Ministry of Public Health, Nonthaburi, 11000 Thailand; 8 Bureau of General Communicable Diseases, Department of Disease Control, Ministry of Public Health, Nonthaburi, 11000 Thailand; 9 Department of Microbiology, Faculty of Medicine Siriraj Hospital, Mahidol University, Bangkok, 10700 Thailand

**Keywords:** Hand foot and mouth disease, Enterovirus 71, Coxsackievirus A16, Seroprevalence

## Abstract

**Background:**

Hand, foot and mouth disease (HFMD) is endemic among population of young children in Thailand. The disease is mostly caused by enterovirus 71 (EV71) and coxsackievirus A16 (CA16).

**Methods:**

This study conducted serosurveillance for neutralizing (NT) antibodies to EV71 subgenotypes B5 and C4a, and to CA16 subgenotypes B1a and B1b, in 579 subjects of various ages using a microneutralization assay in human rhabdomyosarcoma (RD) cells. These test viruses were the major circulating subgenotypes associated with HFMD in Thailand during the study period.

**Results:**

We found that the levels of seropositivity against all 4 study viruses were lowest in the age group of 6–11 months, i.e., 5.5% had antibody to both EV71 subgenotypes, while 14.5% and 16.4% had antibody to CA16 subgenotypes B1a and B1b, respectively. The percentages of subjects with antibodies to these 4 viruses gradually increased with age, but were still less than 50% in children younger than 3 years. These laboratory data were consistent with the epidemiological data collected by the Ministry of Public Health which showed repeatedly that the highest number of HFMD cases was in children aged 1 year. Analyses of amino acid sequences of the test viruses showed 97% identity between the two subgenotypes of EV71, and 99% between the two subgenotypes of CA16. Nevertheless, the levels of seropositivity and antibody titer against the two subgenotypes of EV71 and of CA16 were not significantly different.

**Conclusions:**

This study clearly demonstrated NT antibody activity across EV71-B5 and EV71-C4a subgenotypes, and also across CA16-B1a and CA16-B1b subgenotypes. Moreover, there were no significant differences by gender in the seropositive rates and antibody levels to any of the 4 virus subgenotypes.

**Electronic supplementary material:**

The online version of this article (10.1186/s12985-018-1074-8) contains supplementary material, which is available to authorized users.

## Background

Hand, foot and mouth disease (HFMD) is common in children below the age of 5. The disease is characterized by acute fever with vesicular rashes or blisters on the palms, soles, and oral mucosa. HFMD can be caused by any of several serotypes of human enteroviruses, most commonly enterovirus 71 (EV71) and coxsackievirus A16 (CA16). The co-circulation of EV71 and CA16 in the same season has been observed in many countries [1–3]. CA16-associated HFMD was first reported in Canada in 1957 [4], and that associated with EV71 was first reported in California in 1969 [5]. At present, HFMD is recognized as an endemic disease in the Asia Pacific region. Outbreaks of EV71-associated HFMD have been reported in many countries in Asia, especially in Japan, the People’s Republic of China, Taiwan, Singapore, Vietnam and Cambodia [6–8]. HFMD is usually mild and self-limited, although EV71 might be severe and cause neurological and/or cardiopulmonary complications which may lead to death [9, 10]. Outbreaks of CA16-associated HFMD have been reported in many countries, but the virus mostly causes mild disease [11–13]. Between 2008 and 2015, CA6 became the predominant causative agent of several HFMD outbreaks in a wide geographical area, including Finland, Singapore, Vietnam, China, Taiwan, Japan and Thailand [14–20]. CA6 was less likely to be associated with severe HFMD than EV71. At present, the incidence of CA6 remains significant.

HFMD was recognized as an emerging disease after an EV71-associated outbreak in Malaysia in 1997 which involved approximately 2628 cases with 29 deaths [21]. In 1998, a larger outbreak of HFMD/herpangina occurred in Taiwan and involved approximately 129,106 cases with 78 deaths [22]. Interestingly, no epidemiological linkage between these two outbreaks was identified. Since 2001, HFMD has been listed in Thailand as a disease requiring notification to the Ministry of Public Health (MOPH) [23, 24]. At present, HFMD is recognized as endemic in Thailand. The number of cases reported to the MOPH has markedly increased each year, a trend which is probably due to growing awareness of the disease. The incidence of HFMD reaches its peak in rainy season, between June and September; EV71 and CA16 are the two leading causes and co-circulate.

EV71 and CA16 serotypes belong to Family *Picornaviridae*, Genus *Enterovirus,* species Enterovirus A. An average genome of picornavirus is about 7500 nucleotides long and encodes for a polyprotein which is cleaved into 4 functional structural proteins: VP1, VP2, VP3 and VP4 of the viral capsid. VP1 is immunodominant and functions as the principal neutralizing (NT) domain. VP2 and VP3 also induce NT antibodies, but VP4 does not [25]. As NT antibodies are protective, subjects with NT antibodies will be immune to subsequent infections by related picornaviruses. Based on the VP1 region, EV71 is classified into 6 genotypes: A, B, C, D, E and F [26, 27]. Genotype A is the EV71 prototype and comprises only one member, BrCr. Genotype B is further divided into 5 subgenotypes: B1, B2, B3, B4 and B5; likewise, genotype C into C1, C2, C3, C4a and C4b. Genotypes D, E and F were identified in India and Africa, and are not subdivided [26, 27]. Similarly, based on VP1, CA16 is divided into 3 subgenotypes: A, B1a and B1b; if based on VP4, CA16 is divided into 3 subgenotypes: A, B and C [1, 28]. These distinct subgenotypes are distributed in different geographical areas. A subgenotype may circulate for a period of time after emerging and then fade away over time. An example is subgenotype C4b which was introduced into Thailand in 2006 and disappeared in 2008. At present, the situation in Thailand is similar to that in Taiwan and Singapore where B5 and C4a co-circulate, but in Thailand B5 is the predominant subgenotype.

Several epidemiological studies demonstrated NT antibody to only one subgenotype of EV71 and/or CA16 [15, 29–32], while few reported NT antibody across multiple subgenotypes [33, 34]. This prospective seroepidemiological study aimed to determine the frequency of NT antibodies against subgenotypes of EV71 (B5 and C4a) and CA16 (B1a and B1b) in people of various age-groups in Nakhon Ratchasima Province situated in the northeast of Thailand using a cytopathic effect (CPE)-based microneutralization (MN) assay on rhabdomyosarcoma (RD) cell monolayers. In addition, VP1-VP4 amino acid sequences of the test viruses were analyzed to assess antigenic diversity. This information will be useful for understanding the viral antigenic diversity which is important for vaccine development or vaccine selection for a country.

## Methods

### Ethical issues

This study was approved by two Ethical Committees: the Siriraj Institutional Review Board, Faculty of Medicine Siriraj Hospital, Mahidol University and the Ministry of Public Health Review Board, Nakhon Ratchasima Province. Adult subjects or parents of the child subjects signed the consent form for participation in the study.

### Study site

Nakhon Ratchasima Province is 259 km from Bangkok. It is the major gateway to the northeast of Thailand. In 2013, this province had a population of 2.6 million, with the second highest population next to Bangkok. Based on data from the Bureau of Epidemiology, MOPH, Thailand, this province had the highest incidence of HFMD cases among the northeast provinces, and ranked third in incidence for all of Thailand. [24].

### Test sera

A total of 579 serum samples were collected from subjects who lived in Nakhon Ratchasima Province in 2013. These subjects ranged in age from < 1 to 60 years old. Samples from children under 5 were the leftover sera from non-HFMD cases after routine investigations for disease diagnosis/treatment in hospitals located in various districts of the province. Sera from those of age older than 5 years were collected from healthy subjects in several communities. The test sera were aliquoted and stored at − 20 °C until used.

### Test viruses

The test viruses used in MN assays for NT antibodies were: two strains of EV71, SiICRC10/TH/2011 subgenotype B5 (EV71-B5/2011) and SiICRC01/TH/2014 subgenotype C4a (EV71-C4a/2014); two strains of CA16, SiICRC06/TH/2011 subgenotype B1a (CA16-B1a/2011) and SiICRC01/TH/2012 subgenotype B1b (CA16-B1b/2012). These viruses were isolated from clinical specimens from HFMD patients in Vero cells (African green monkey kidney cells - ATCC, CCL-81) and propagated in RD cells (derived from human rhabdomyosarcoma). Both Vero and RD cell lines were grown in Eagle’s minimum essential medium (EMEM) (Gibco, NY) supplemented with 10% fetal bovine serum (FBS) (Gibco); infected RD cell monolayers were maintained in EMEM supplemented with 2% FBS. The complete VP1 regions of these virus isolates were sequenced and phylogenetically analyzed for genotypic and subgenotypic identification. The virus titers were determined in RD cells and expressed as 50% tissue culture infectious dose (TCID50) according to the Reed-Muench method [35]. The test viruses were aliquoted and stored at − 80 °C until used.

### Microneutralization assay

The CPE-based MN assay was conducted in 96-well micro-culture plates. Each test serum was heat inactivated at 56 °C for 30 min and serially two-fold diluted with maintenance medium starting from a dilution of 1:10 to 1:1280. The assay was performed by mixing 60 μl of the diluted serum with 60 μl of the test virus suspension at a concentration of 200 TCID50 and incubated at 37 °C for 2 h. Then, a volume of 100 μl of the virus-serum mixture was transferred into a well of RD cell monolayer and further incubated at 37 °C for 5–6 days. In order to verify the amount of virus inoculum at the concentration of 100 TCID50, the virus back-titration at doses of 0.1, 1, 10, and 100 TCID50 were included in every assay plate, together with positive control with known antibody titer and cell culture controls. Test reactions were run in duplicate. The inoculated cell monolayers were observed for appearance of CPE indicating viral infection. The antibody titer was defined as the reciprocal of the highest serum dilution that protected 50% of the inoculated cell monolayers from the virus infection, i.e., the last dilution of NT antibody-positive serum which exhibited ≤2+ degrees of CPE. Test sera with NT antibody titers of ≥10 were considered seropositive. To calculate the geometric mean titer (GMT), sera with NT antibody titers of < 10 were assigned a value of 5 and sera with NT antibody titers of ≥1280 were assigned a value of 1280.

### Viral nucleotide sequencing

Total RNAs were extracted from the virus suspensions using the QIAamp Viral RNA (QIAGEN, Venlo, Netherlands). The extracted RNAs were used as targets for amplification of VP1 to VP4 regions by reverse transcription-polymerase chain reaction (RT-PCR) using the One Step RT-PCR kit (QIAGEN). Briefly, a total 50 μl volume for RT-PCR consisted of 10 μl of RNA template in master mix containing 1X RT-PCR buffer, 2 μl of enzyme mix, 2 μl of dNTP mix, 5 units of RNase inhibitor (Promega Corporation, Madison, WI), and 400 nM of primers. The reaction was carried out in a thermal cycler (GeneAmp PCR system 2400, Applied Biosystems, Foster City, CA). The PCR cycling program consisted of one cycle at 50 °C for 30 min and one cycle at 95 °C for 15 min, followed by 35 cycles at 94 °C for 30 s, 55 °C for 30 s, 72 °C for 60 s and a final extension step cycle at 72 °C for 10 min. The amplified product was electrophoresed in 1.5% agarose gel in TAE buffer and purified using a QIAEX II gel extraction kit (QIAGEN). The primer sets, shown in an Additional file 1 (Table S1), were designed by our group and used for both RT-PCR and nucleotide sequencing. The purified PCR products were sequenced at Macrogen Korea. The nucleotide sequences were edited and aligned using Bioedit version 7.2.6.1.

## Results

### Prevalence of HFMD, and EV71 and CA16 subgenotypes in Thai children

**Fig. 1:**
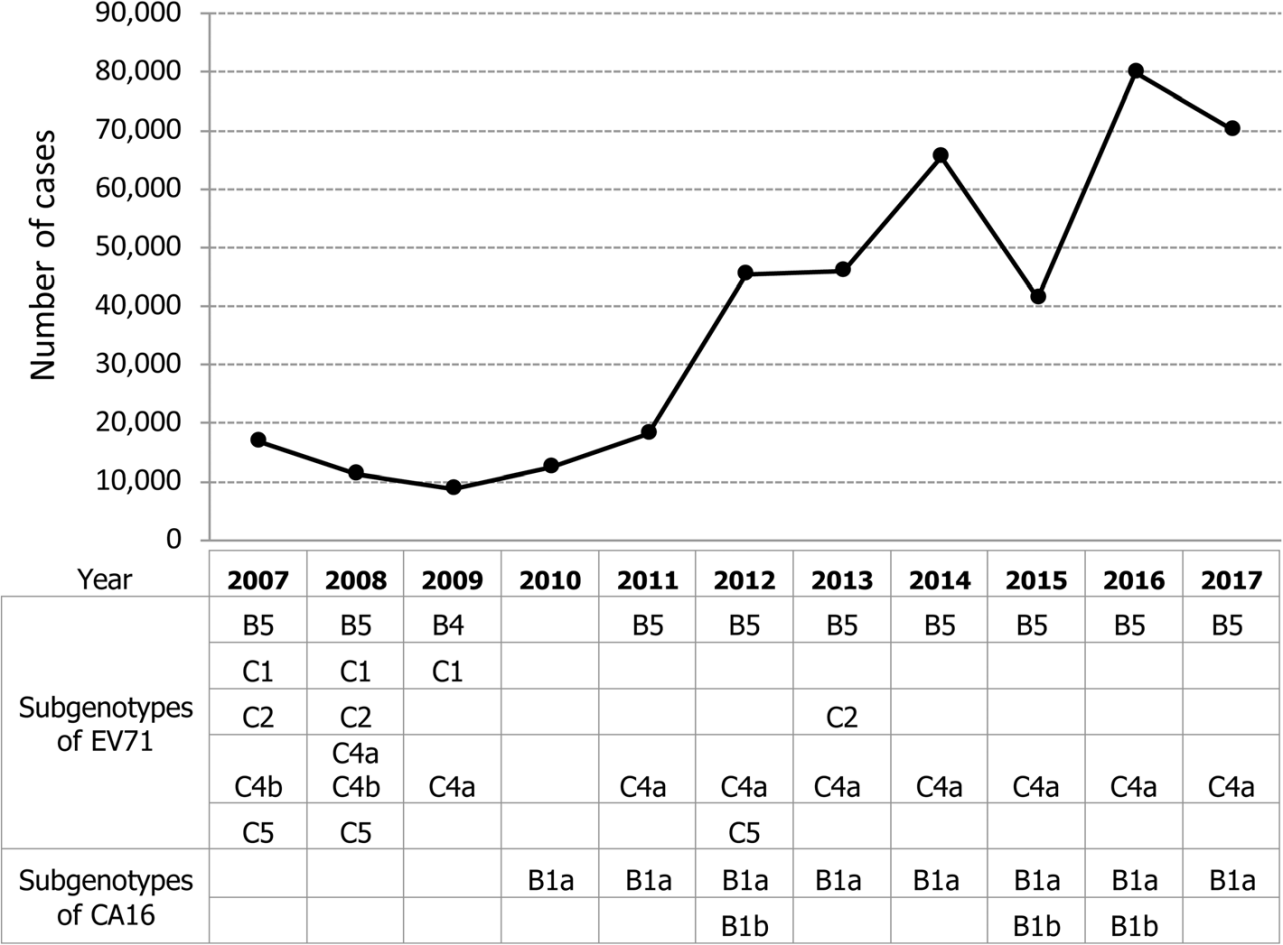
Number of HFMD cases reported annually by Bureau of Epidemiology, MOPH, Thailand during 2007–2017. Subgenotypes of EV71 and CA16 are identified by our analysis based on sequencing data from our group, the National Institute of Health, Department of Medical Sciences, MOPH, and the GenBank database

According to a report from the Bureau of Epidemiology (BOE), Department of Disease Control, MOPH, from 2007 to 2017, HFMD occurred commonly in children less than 5 years of age (Additional file 2: Figure S1). Throughout the last decade, the highest number of cases was repeatedly observed in children aged one year and gradually declined with older age. The number of HFMD cases reported to the MOPH rose from 10,000–20,000 cases in 2007–2011 to 70,000–80,000 in 2016–2017. Based on complete VP1 sequences, various subgenotypes of EV71 and CA16 were identified during the study period (2013) by both our laboratory and that of the National Institute of Health, Department of Medical Sciences, MOPH (Fig. 1).

### Cross-neutralizing antibody against EV71 or CA16 subgenotypes by age and sex

**Fig. 2:**
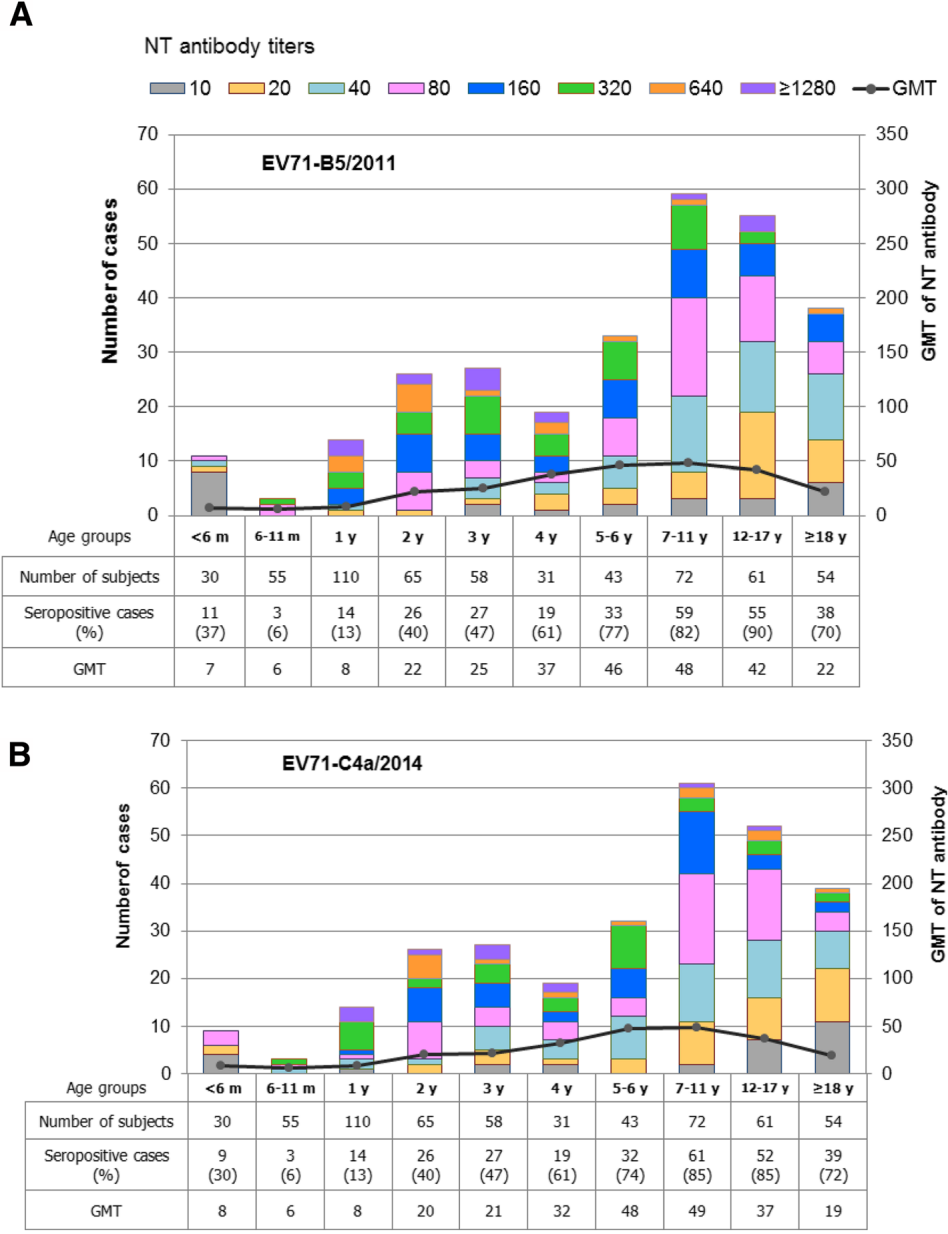
Distribution of NT antibody titers and GMTs against EV71-B5 (**a**) and EV71-C4a (**b**) by age-group

**Fig. 3:**
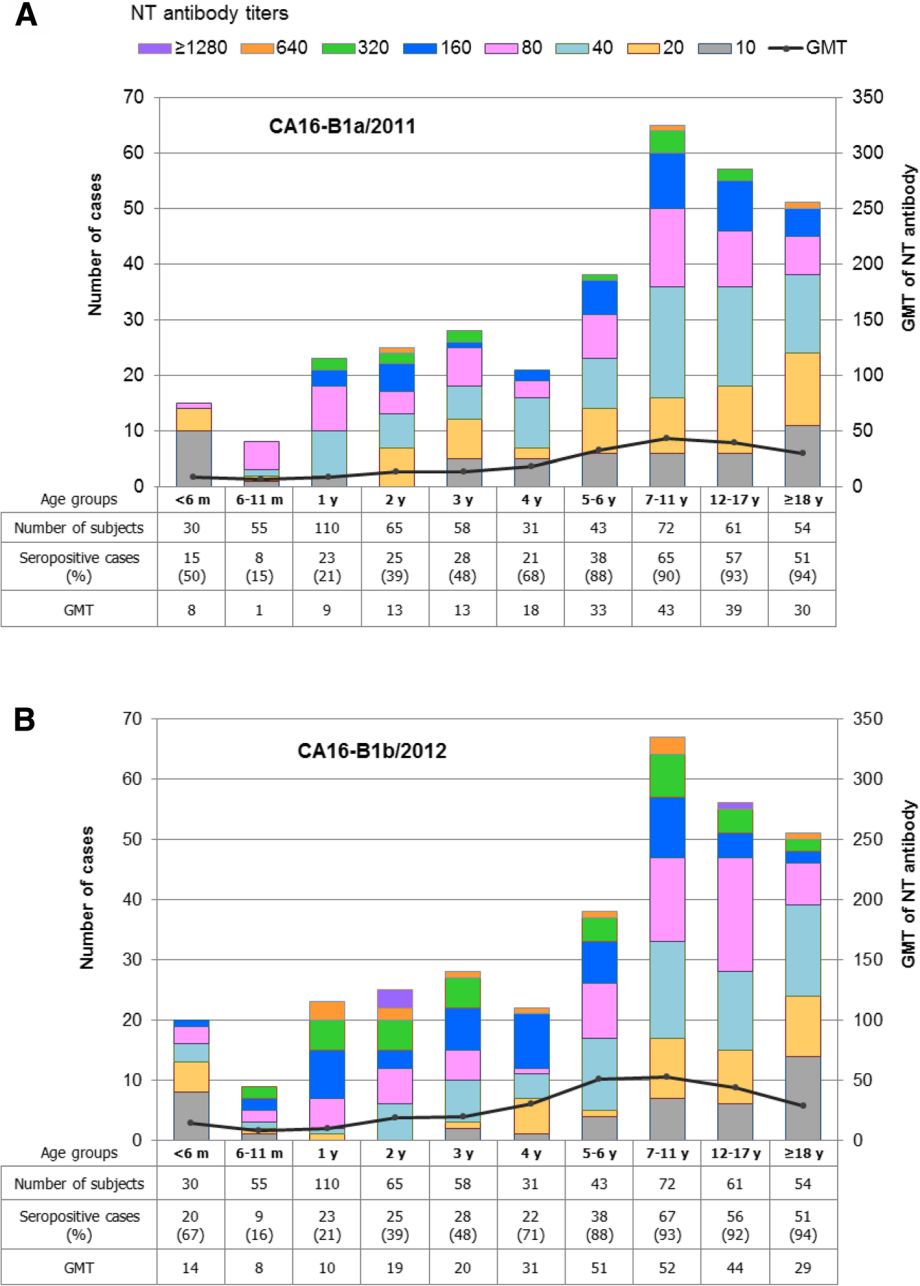
Distribution of NT antibody titers and GMTs against CA16-B1a (**a**) and CA16-B1b (**b**) by age-group

Sera from a total of 579 subjects from various age-groups were titrated for presence of NT antibodies to EV71-B5, EV71-C4a, CA16-B1a and CA16-B1b at the initial serum dilution of 1:10 using a CPE-based MN assay in RD cells. The presence of NT antibody detected in infants younger than 6 months of age was not accepted as a true result because their sera might contain maternally transferred antibody. Antibody responses to natural infection should account for results in the older age-groups. Distribution of NT antibody titers and GMTs against the 4 subgenotypes in various age-groups among the study subjects are shown in Figs. 2 and 3. The seropositive rates against all 4 study viruses were lowest in the children aged 6–11 months, i.e., 5.5% had antibody to both subgenotypes of EV71, and 14.5% and 16.4% to CA16 subgenotypes B1a and B1b, respectively. The number of subjects with antibodies to these 4 viruses gradually increased with age, but was still less than 50% in children of age less than 3 years.

**Fig. 4:**
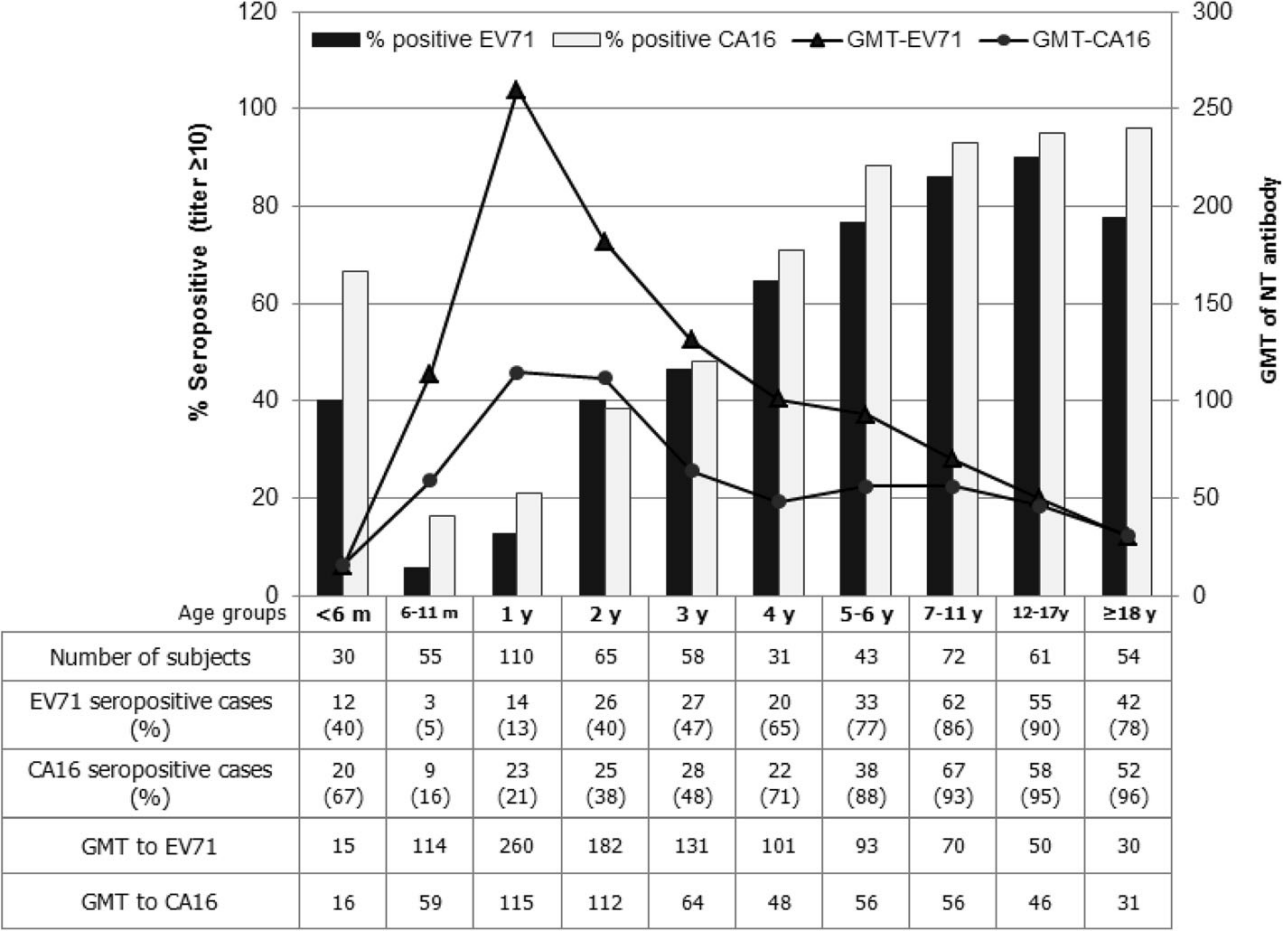
Seroprevalence of NT antibodies to EV71 and CA16 by age-group, irrespective of the virus subgenotypes. GMTs are based on only the positive serum samples

The EV71 seropositive rates rose to peaks is the age-group 12–17 years (90.2% for antibody to EV71-B5 and 85.2% to EV71-C4a), and then declined (Fig. 2a and b). There were neither significant differences in seropositivity (Mann-Whitney U test, *P* > 0.05) nor antibody levels (paired t-test; *P* > 0.05) between the B5 and C4a subgenotypes. The seropositive rates of NT antibody to CA16 were slightly higher than those to EV71. Percentages of subjects with NT antibody to CA16-B1a or CA16-B1b were greater than 90% in the age-groups of 7–11 years and older (Fig. 3a and b). There were no significant differences in seropositivity (Mann-Whitney U test, *P* > 0.05) and antibody levels (paired t-test; *P* > 0.05) between the B1a and B1b subgenotypes. Our study clearly demonstrated the presence of NT antibody activity to the EV71-B5 and EV71-C4a subgenotypes, and also to the CA16-B1a and CA16-B1b subgenotypes. After merging the data sets, the seroprevalence of EV71 and CA16 NT antibodies in various age-groups, irrespective of subgenotypes, is shown in Fig. 4. Collectively, the study implied that the age-groups younger than 5 years were vulnerable to both EV71 and CA16 infections. A notable feature is that the percent seropositivity to both EV71 and CA16 exceeded those to EV71 or CA16 alone, and that this dual positivity progressively increased from age of 3 years onwards. Greater than 80% of the subjects in the age-groups older than 7–11 were immune to both serotypes (Table 1).

**Table 1:** Seropositivity of NT antibody to EV71 and/or CA16 by age groups

Age	*N*	% seropositive		
		+ve both EV71 and CA16	+ve EV71 only	+ve CA16 only
< 6 m	30	33.3	6.7	33.3
6–11 m	55	0.0	5.5	16.4
1 y	110	0.9	11.8	20.0
2 y	65	16.9	23.1	21.5
3 y	58	27.6	19.0	20.7
4 y	31	41.9	22.6	29.0
5–6 y	43	67.4	9.3	20.9
7–11 y	72	80.6	5.6	12.5
12–17 y	61	86.9	3.3	8.2
≥18 y	54	74.1	3.7	22.2
Total (All age groups)	579	39.9	10.9	19.2

The total of 579 subjects in this study consisted of 285 males and 294 females (male to female sex ratio of 1:0.97). There were no statistically significant gender-based differences in the number of seropositive subjects (Chi-square test, *P* > 0.05) nor their GMTs (Mann-Whitney U test, *P* > 0.05) to any of the 4 test viruses (Additional file 3: Figure S2).

### Amino acid identity between subgenotypes

Amino acid sequences in the VP1, VP2, VP3 and VP4 regions of EV71-B5 were aligned against those of EV71-C4a, and those of CA16-B1a against CA16-B1b. The results showed an identity of 97% among 5 strains of the two subgenotypes of EV71, and 99% among 4 strains of the two subgenotypes of CA16 (Additional file 4: Figure S3 and Additional file 5: Figure S4). However, the amino acid sequences of VP1-VP4 of the two CA16 subgenotypes used in this study were 100% identical.

## Discussion

HFMD is an uncommon disease in adults, while it is common in very young children. The rarity in adults is probably due to protective immunity induced by previous natural infections. In fact, low seropositive rates are inversely correlated with high prevalences of HFMD in young age-groups. The present study showed that 30–37% of children aged < 6 months were EV71 seropositive, and the seropositive rates decreased to 6% in the age-group of 6–11 months and 13% in the age-group of 1 year. Similarly, the prevalence of NT antibody against CA16 was 50–67% in children aged < 6 months, and then decreased to 15–16% in the age-group of 6–11 months and increased to 21% in the age-group of 1 year. The higher prevalence of enteroviral antibodies in children younger than 6 months is suggestive of maternally transferred antibody which rapidly declined in the older age-groups. A meta-analysis by other investigators also found that maternal antibody waned almost completely by 5 months of age [32].

The prevalence of enteroviral antibodies varies by country, but the results of this study were similar to those reported previously by investigators from Thailand [36], Singapore [37], Russia [38] and Xiamen City, China [39]. Overall, those data show that the lowest levels of seropositivity (5–43%) are found in children aged 1–2 years, and rises to peak between 7 and 19 years of age. The serological data from Cambodia is obviously different from the others as the seropositivity in children of age > 2 years in 2006–2011 was higher than 90% and, despite that, a serious EV71-associated outbreak occurred in 2012 and involved at least 113 severe cases with 54 deaths [31]. Interestingly, EV71 infection was less common in Singapore. A study of serum samples collected during 2008 to 2010 from subjects aged 1–17 years showed that the seroprevalence of CA6 NT antibody was highest (62.7%), followed by CA16 (60.6%) and EV71 (29.3%) [15]. Approximately 50% of children in the age-group of 1–6 years had antibody to CA6 or CA16, while only 15% had antibody to EV71. This is consistent with a report that antibody induced by inactivated EV71 does not cross protect against CA16 and vice versa [40].

The differences in levels of seropositivity and GMT reported from various studies could not be explained by the differences in study populations or geographic location alone. As there is no standard protocol recommended for the MN assay, laboratory techniques and reagents employed in different laboratories are also different. Due to these differences, NT antibody titers may vary and result in some difference in the calculated seroprevalence rates.

Our important findings were that the lowest levels of seropositivity of NT antibodies to EV71 and CA16 were in children of age less than 1 year, and that less than 50% of children of age younger than 3 years were seropositive. These findings are consistent with the epidemiological data from the MOPH, Thailand, such that most HFMD cases occurred in children aged 1 year or less, followed by those of age less than 2 and 3 years, respectively. Our results suggested that children of age under 3 years were at high risk to get EV71 or CA16 infection. In particular, those in the age-group of 6–11 months were the most vulnerable to contract EV71- or CA16-associated HFMD. Among children older than 7–11 years, ≥80% were immune to both viruses.

There was no difference in seropositivity to EV71 and CA16 by gender found in this study nor that of others [31, 41, 42]. However, a significant difference by gender in the prevalence of antibody to EV71, but not to CA16, is reported from China [29, 30].

It is reported that VP1, VP2 and VP3 capsid proteins all contain neutralizing epitopes, but that VP1 contains the major neutralizing domain [43, 44]. Our analysis on intra-subgenotypic variation demonstrated that there were 3 amino acid differences (position 239 in VP2, and positions 663 and 857 in VP1) among 3 strains within the B5 subgenotype; and there were 4 positions of difference between 2 strains of the C4a subgenotype (positions 545 and 557 in VP3, and positions 805 and 814 in VP1). Regarding inter-subgenotypic variation between EV71-B5 and C4a subgenotypes, a total of 24 amino acid differences were found in VP1-VP3, but none in VP4 as shown in (Additional file 4: Figure S3). Nevertheless, the seropositivity and antibody levels, as determined with these two EV71 subgenotypes, were not significantly different. Similar results were also obtained in this study with CA16-B1a and B1b subgenotypes. Our analysis for the intra-subgenotypic variation demonstrated that there were 3 amino acid differences (position 286 in VP2, and positions 729 and 816 in VP1) in 2 strains within the B1a subgenotype; and there were 2 positions of difference between 2 strains of the B1b subgenotype (positions 53 in VP4 and 588 in VP1). Regarding inter-subgenotypic variation among the 4 strains of CA16-B1a and B1b subgenotypes, a total of 5 amino acid differences were found in VP1, VP2, and VP4, but none in VP3 as shown in (Additional file 5: Figure S4). However, the CA16 subgenotypes B1a and B1b used as the test antigens in this study showed the identities of 91.2% for nucleotide sequence and 100% for amino acid sequence of the VP1-VP4 regions. Along the entire genomes of these two CA16 subgenotypes, the difference in amino acid sequence of 1.6% was located in non-structural regions only (data not shown). As such, the significant difference in the level of NT antibodies against these two CA16 subgenotypes in this study was not observed.

EV71 subgenotypic replacement has been shown in different countries during the past 20 years [27, 45], including Thailand where subgenotypes B5 and C4a have cocirculated for longer than 5 years. Subgenotypic replacement becomes an important issue in the context of vaccine development. Different EV71 strains were used for vaccine production by different manufacturers. A vaccine strain that induces NT antibody which can react across subgenotypes is desirable. EV71 vaccine strains used by 3 manufacturers in mainland China belong to subgenotype C4a, while that developed in Taiwan belongs to B4 and that in Singapore to B3 [46]. The results show that NT antibody induced by a C4a vaccine cross-neutralize EV71 subgenotypes B3, C2, C3, and C5 but not subgenotype A [47]; the NT antibody induced by a B4 vaccine strongly cross-neutralize B1, B5 and C4a, but poorly neutralize C4b [48]. The information above demonstrates that the relatedness between the vaccine strain and the circulating strain(s) should be considered for vaccine selection in each country. NT antibodies which react across EV71 subgenotypes are also observed in patients with HFMD [34, 49]. It is noted that the cross reactive antibodies in those studies were demonstrated in the vaccinees and HFMD patients. In contrast, our study showed that cross-reactive NT antibody activity against EV71 subgenotypes B5 and C4a existed in the general population.

The collective information from different groups of investigators suggests that protection conferred by EV71 vaccines across subgenotypes may be strain/subgenotype specific. As subgenotypic replacement is common for EV71, evaluation of the efficacy of EV71 vaccines should take account of the circulating strains in each geographical area in each year.

## Conclusions

This study demonstrated that the lowest levels of seropositivity of NT antibodies to EV71 and CA16 were in children of age less than 1 year, and that less than 50% of children of age younger than 3 years were seropositive. Therefore, children of age younger than 1 year were the most vulnerable to both EV71 and CA16 infections, followed by children of age 2 and 3 years, respectively. These findings are consistent with the epidemiological data from the MOPH, Thailand. We emphasize the importance of serosurveillance to estimate the risk of HFMD outbreaks in child populations. This data also will be useful for vaccine strain selection in the near future.

## Additional files

Additional file 1: **Table S1.** The primer sets for amplification and nucleotide sequencing VP1-VP4 of EV71 and CA16. (PDF 147 kb)

Additional file 2: **Figure S1.** Age-related HFMD cases in Thailand during 2007–2017. A summary graph of the data reported by the Bureau of Epidemiology, MOPH, Thailand. (PDF 242 kb)

Additional file 3: **Figure S2.** Seropositivity of NT antibodies against EV71 (A) and CA16 (B) by gender. (PDF 393 kb)

Additional file 4: **Figure S3.** VP1-VP4 amino acid alignment of EV71. (PDF 183 kb)

Additional file 5: **Figure S4.** VP1-VP4 amino acid alignment of CA16. (PDF 250 kb)

## Abbreviations

BOE: Bureau of Epidemiology
CA16: Coxsackievirus A16
CPE: Cytopathic effect
EMEM: Eagle’s minimum essential medium
EV71: Enterovirus 71
FBS: Fetal bovine serum
GMT: Geometric mean titer
HFMD: Hand, foot and mouth disease
MN: Microneutralization
MOPH: the Ministry of Public Health
NT: Neutralizing
RD: Rhabdomyosarcoma
TCID50: 50% tissue culture infectious dose
