# Supplementary material for: Seroprevalence of antibodies to enterovirus 71 and coxsackievirus A16 among people of various age groups in a northeast province of Thailand
Source: Virol J. 2018 Oct 16;15:158. doi: 10.1186/s12985-018-1074-8 (PMC6192276; doi:10.1186/s12985-018-1074-8)
Supplement: Supplementary file 1 — Table S1. The primer sets for amplification and nucleotide sequencing VP1-VP4 of EV71 and CA16. (PDF 147 kb) [file 12985_2018_1074_MOESM1_ESM.pdf]

Table S1 The primer sets for amplification and nucleotide sequencing VP1-VP4 of EV71 and CA16

Fragment	Primers	Position	Product size (bp)	Sequences (5'---3')
	**Primer sets of EV71**			
1	FL-F-1-C	1 - 21	~1,075	TTAAAACAGCCTGTGGGTTG
	1075R	1061-1080		CAGTAMGAYGGCCARTCKCC
	636R*	619-639		CCAATCCAATAGCTATATGGC
2	447F	447 - 467	~920	TAGTCCTCCGGCCCCTGAAT
	1366R	1349-1367		CGCCTGCCACTGTCCCWAT
3	1178F	1181- 1198	~1,200	TCCAAGGGRTGGTAYTGG
	2376R	2357- 2376		GCYGCCGCYAGTGCTATTAT
4	2191F	2192- 2210	~1,255	TTTGGGCTRCAATCRTCTG
	3446R	3428-3446		GRGAGCTGTCTTCCCAAAC
	**Primer sets of CA16**			
1	CA16_A	1- 20	~1,075	TTAAAACAGCCTGTGGGTTG
	CA16_A2R	1057-1074		CCAYTCCCCATAKGCTAT
	CA16_A1R*	592 - 611		CAATTGTCACCATAAGCAGC
2	CA16_B1F	543-560	~1,140	GCGGAACCGACTACTTTG
	CA16_B1R	1666-1683		RCACATGGGAGCTATGGT
3	CA16_B2F	1423-1440	~1,014	CAGGTTGGTGCAGTHCT
	CA16_B2R	2417-2435		GTTTGCTCAATRTCCTCHG
4	CA16_B3F	2182-2220	~1,006	CATGTGATCTGGGACTTY
	CA16_B3R	3169-3188		ATGGAGTGTGGTGACTTCTC

VP1-VP4 regions of EV71 and CA16 were amplified into 4 fragments of DNA products by using 4 pairs of primer sets; all these primers were also used as sequencing primers.

*Primers 636R and CA16_A1R were only used as sequencing primers for fragment 1 of EV71 and CA16, respectively.
