# Supplementary material for: Seroprevalence of antibodies to enterovirus 71 and coxsackievirus A16 among people of various age groups in a northeast province of Thailand
Source: Virol J. 2018 Oct 16;15:158. doi: 10.1186/s12985-018-1074-8 (PMC6192276; doi:10.1186/s12985-018-1074-8)
Supplement: Supplementary file 2 — Figure S1. Age-related HFMD cases in Thailand during 2007–2017. A summary graph of the data reported by the Bureau of Epidemiology, MOPH, Thailand. (PDF 242 kb) [file 12985_2018_1074_MOESM2_ESM.pdf]

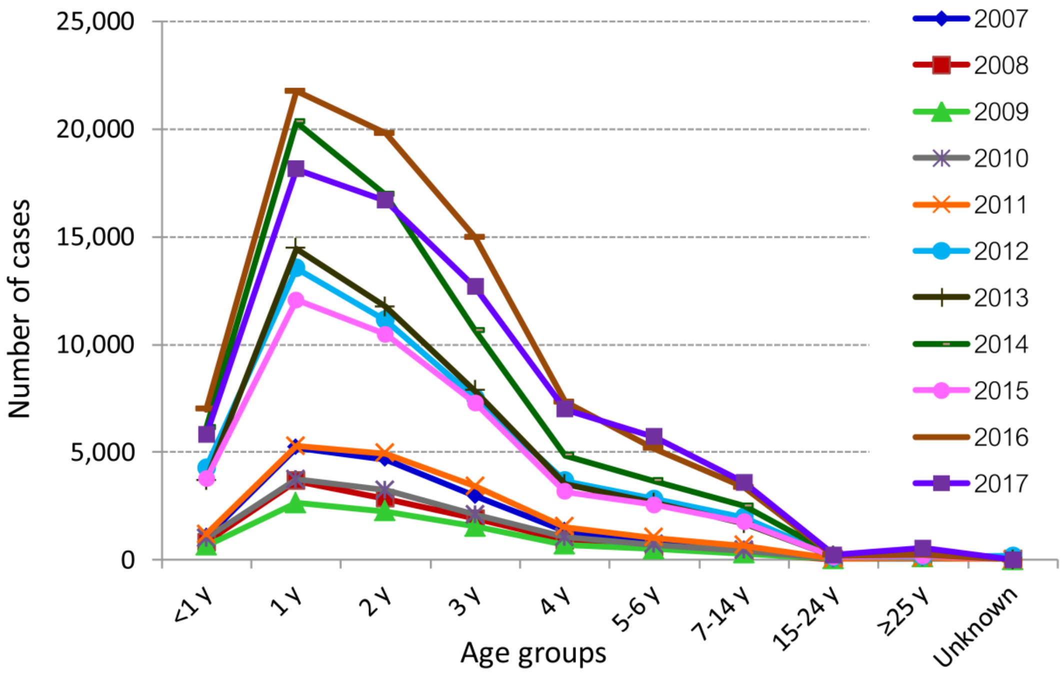

Figure S1 Age-related HFMD cases in Thailand during 2007-2017. A summary graph of the data reported by the Bureau of Epidemiology, MOPH, Thailand.
