# Supplementary figures and images for: Seroprevalence of antibodies to enterovirus 71 and coxsackievirus A16 among people of various age groups in a northeast province of Thailand
Source: Virol J. 2018 Oct 16;15:158. doi: 10.1186/s12985-018-1074-8 (PMC6192276; doi:10.1186/s12985-018-1074-8)

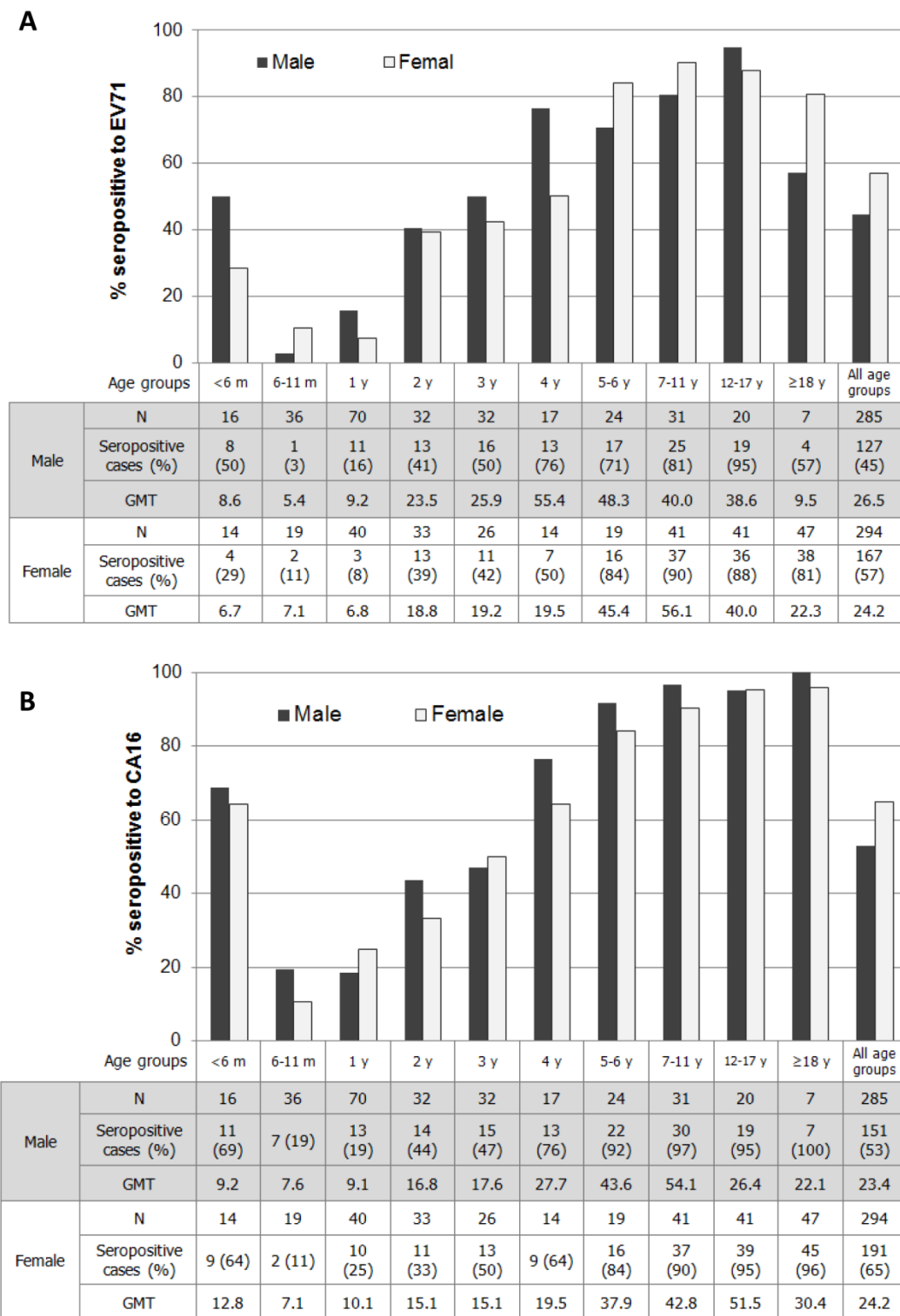

**Figure S2** Seropositivity of NT antibodies against EV71 (A) and CA16 (B) by gender.

Supplement: Supplementary file 3 — Figure S2. Seropositivity of NT antibodies against EV71 (A) and CA16 (B) by gender. (PDF 393 kb) [file 12985_2018_1074_MOESM3_ESM.pdf]
