# Supplementary material for: Seroprevalence of antibodies to enterovirus 71 and coxsackievirus A16 among people of various age groups in a northeast province of Thailand
Source: Virol J. 2018 Oct 16;15:158. doi: 10.1186/s12985-018-1074-8 (PMC6192276; doi:10.1186/s12985-018-1074-8)
Supplement: Supplementary file 4 — Figure S3. VP1-VP4 amino acid alignment of EV71. (PDF 183 kb) [file 12985_2018_1074_MOESM4_ESM.pdf]

```
                                  VP4 (1-69)
                                          10         20         30         40         50         60
                                  ....|....| ....|....| ....|....| ....|....| ....|....| ....|....|
SiICRC10/TH/2011(EV71-B5)**       MGSQVSTQRS GSHENSNSAT EGSTINYTTI NYYKDSYAAT AGKQSLKQDP DKFANPVKDI
SiICRC05/TH/2014(EV71-B5)         .......... .......... .......... .......... .......... ..........
SiICRC07/TH/2014(EV71-B5)         .......... .......... .......... .......... .......... ..........
SiICRC01/TH/2014(EV71-C4a)**      .......... .......... .......... .......... .......... ..........
TH/MU-1/2015(EV71-C4a)            .......... .......... .......... .......... .......... ..........

                                             VP2 (70-323)
                                          70         80         90        100        110        120
                                  ....|....| ....|....| ....|....| ....|....| ....|....| ....|....|
SiICRC10/TH/2011(EV71-B5)**       FTEMAAPLKS PSAEACGYSD RVAQLTIGNS TITTQEAANI IVGYGEWPSY CSDDDATAVD
SiICRC05/TH/2014(EV71-B5)         .......... .......... .......... .......... .......... ..........
SiICRC07/TH/2014(EV71-B5)         .......... .......... .......... .......... .......... ..........
SiICRC01/TH/2014(EV71-C4a)**      .......... .......... .......... .......... .......... ...S......
TH/MU-1/2015(EV71-C4a)            .......... .......... .......... .......... .......... ...S......

                                         130        140        150        160        170        180
                                  ....|....| ....|....| ....|....| ....|....| ....|....| ....|....|
SiICRC10/TH/2011(EV71-B5)**       KPTRPDVSVN RFYTLDTKLW EKSSKGWYWK FPDVLTETGV FGQNAQFHYL YRSGFCIHVQ
SiICRC05/TH/2014(EV71-B5)         .......... .......... .......... .......... .......... ..........
SiICRC07/TH/2014(EV71-B5)         .......... .......... .......... .......... .......... ..........
SiICRC01/TH/2014(EV71-C4a)**      .......... .......... .......... .......... .......... ..........
TH/MU-1/2015(EV71-C4a)            .......... .......... .......... .......... .......... ..........

                                         190        200        210        220        230        240
                                  ....|....| ....|....| ....|....| ....|....| ....|....| ....|....|
SiICRC10/TH/2011(EV71-B5)**       CNASKFHQGA LLVAILPEYV IGTVAGGTGT EDSHPPYKQT QPGADGFELQ HPYVLDAGVP
SiICRC05/TH/2014(EV71-B5)         .......... .......... .......... .......... .......... ........I.
SiICRC07/TH/2014(EV71-B5)         .......... .......... .......... .......... .......... ........I.
SiICRC01/TH/2014(EV71-C4a)**      .......... ....V..... .......... ..T....... .......... ........I.
TH/MU-1/2015(EV71-C4a)            .......... ....V..... .......... ..T....... .......... ........I.

                                         250        260        270        280        290        300
                                  ....|....| ....|....| ....|....| ....|....| ....|....| ....|....|
SiICRC10/TH/2011(EV71-B5)**       ISQLTICPHQ WINLRTNNCA TIIVPYMNTL PFDSALNHCN FGLLVVPISP LDFDQGATPV
SiICRC05/TH/2014(EV71-B5)         .......... .......... .......... .......... .......... ..........
SiICRC07/TH/2014(EV71-B5)         .......... .......... .......... .......... .......... ..........
SiICRC01/TH/2014(EV71-C4a)**      .....V.... .......... ......I.A. .......... .......... ..Y.......
TH/MU-1/2015(EV71-C4a)            .....V.... .......... ......I.A. .......... .......... ..Y.......

                                                           VP3 (324-565)
                                         310        320        330        340        350        360
                                  ....|....| ....|....| ....|....| ....|....| ....|....| ....|....|
SiICRC10/TH/2011(EV71-B5)**       IPITITLAPM CSEFAGLRQA VTQGFPTEPK PGTNQFLTTD DGVSAPILPN FHPTPCIHIP
SiICRC05/TH/2014(EV71-B5)         .......... .......... .......... .......... .......... ..........
SiICRC07/TH/2014(EV71-B5)         .......... .......... .......... .......... .......... ..........
SiICRC01/TH/2014(EV71-C4a)**      .......... .......... ........L. .......... .......... ..........
TH/MU-1/2015(EV71-C4a)            .......... .......... ........L. .......... .......... ..........

                                         370        380        390        400        410        420
                                  ....|....| ....|....| ....|....| ....|....| ....|....| ....|....|
SiICRC10/TH/2011(EV71-B5)**       GEVRNLLELC QVETILEVNN VPTNATSLME RLRFPVSAQA GKGELCAVFR ADPGRDGPWQ
SiICRC05/TH/2014(EV71-B5)         .......... .......... .......... .......... .......... ..........
SiICRC07/TH/2014(EV71-B5)         .......... .......... .......... .......... .......... ..........
SiICRC01/TH/2014(EV71-C4a)**      .......... .......... .......... .......... .......... .....N....
TH/MU-1/2015(EV71-C4a)            .......... .......... .......... .......... .......... .....N....

                                         430        440        450        460        470        480
                                  ....|....| ....|....| ....|....| ....|....| ....|....| ....|....|
SiICRC10/TH/2011(EV71-B5)**       STMLGQLCGY YTQWSGSLEV TFMFTGSFMA TGKMLIAYTP PGGPLPKDRA TAMLGTHVIW
SiICRC05/TH/2014(EV71-B5)         .......... .......... .......... .......... .......... ..........
SiICRC07/TH/2014(EV71-B5)         .......... .......... .......... .......... .......... ..........
SiICRC01/TH/2014(EV71-C4a)**      ..L....... .......... .......... .......... .......... ..........
TH/MU-1/2015(EV71-C4a)            ..L....... .......... .......... .......... .......... ..........
```

Figure S3 VP1-VP4 amino acid alignment of EV71. GenBank accession numbers: KX372311 for SiICRC10/TH/2011(EV71-B5), KX372329 for SiICRC05/TH/2014(EV71-B5), KX372331 for SiICRC07/TH/2014(EV71-B5), KX372328 for SiICRC01/TH/2014(EV71-C4a), and MH879029 for TH/MU-1/2015(EV71-C4a). ** Test viruses for MN assay in this study

```
                                     490        500        510        520        530        540
                                ----|----| ----|----| ----|----| ----|----| ----|----| ----|----|
SiICRC10/TH/2011(EV71-B5)**     DFGLQSSVTL VIPWISNTHY RAHARDGVFD YYTTGLVSIW YQTNYVVPIG APNTAYIIAL
SiICRC05/TH/2014(EV71-B5)       .......... .......... .......... .......... .......... ..........
SiICRC07/TH/2014(EV71-B5)       .......... .......... .......... .......... .......... ..........
SiICRC01/TH/2014(EV71-C4a)**    .......... .......... .......... .......... .......... ..........
TH/MU-1/2015(EV71-C4a)          .......... .......... .......... .......... .......... ..........

                                                         VP1 (566 - 862)
                                     550        560        570        580        590        600
                                ----|----| ----|----| ----|----| ----|----| ----|----| ----|----|
SiICRC10/TH/2011(EV71-B5)**     AAAQKNFTMK LCKDTSHILQ TASIQGDRVA DVIESSIGDS VSRALTRALP APTGQNTQVS
SiICRC05/TH/2014(EV71-B5)       .......... .......... .......... .......... .......... ..........
SiICRC07/TH/2014(EV71-B5)       .......... .......... .......... .......... .......... ..........
SiICRC01/TH/2014(EV71-C4a)**    ....T..... ....A.A... .GT....... .......... ......H... ..........
TH/MU-1/2015(EV71-C4a)          .......... ....A.D... .GT....... .......... ......H... ..........

                                     610        620        630        640        650        660
                                ----|----| ----|----| ----|----| ----|----| ----|----| ----|----|
SiICRC10/TH/2011(EV71-B5)**     SHRLDTGEVP ALQAAEIGAS SNTSDESMIE TRCVLNSHST AETTLDSFFS RAGLVGEIDL
SiICRC05/TH/2014(EV71-B5)       .......... .......... .......... .......... .......... ..........
SiICRC07/TH/2014(EV71-B5)       .......... .......... .......... .......... .......... ..........
SiICRC01/TH/2014(EV71-C4a)**    .......K.. .......... ..A....... .......... .......... ..........
TH/MU-1/2015(EV71-C4a)          .......K.. .......... ..A....... .......... .......... ..........

                                     670        680        690        700        710        720
                                ----|----| ----|----| ----|----| ----|----| ----|----| ----|----|
SiICRC10/TH/2011(EV71-B5)**     PLKGTTNPNG YANWDIDITG YAQMRRKVEL FTYMRFDAEF TFVACTPTGE VVPQLLQYMF
SiICRC05/TH/2014(EV71-B5)       ..E....... .......... .......... .......... .......... ..........
SiICRC07/TH/2014(EV71-B5)       ..E....... .......... .......... .......... .......... ..........
SiICRC01/TH/2014(EV71-C4a)**    ..E....... .......... .......... .......... .......... ..........
TH/MU-1/2015(EV71-C4a)          ..E....... .......... .......... .......... .......... ..........

                                     730        740        750        760        770        780
                                ----|----| ----|----| ----|----| ----|----| ----|----| ----|----|
SiICRC10/TH/2011(EV71-B5)**     VPPGAPKPDS RESLAWQTAT NPSVFVKLTD PPAQVSVPFM SPASAYQWFY DGYPTFGEHK
SiICRC05/TH/2014(EV71-B5)       .......... .......... .......... .......... .......... ..........
SiICRC07/TH/2014(EV71-B5)       .......... .......... .......... .......... .......... ..........
SiICRC01/TH/2014(EV71-C4a)**    .......... .......... ........S. .......... .......... ..........
TH/MU-1/2015(EV71-C4a)          .......... .......... ........S. .......... .......... ..........

                                     790        800        810        820        830        840
                                ----|----| ----|----| ----|----| ----|----| ----|----| ----|----|
SiICRC10/TH/2011(EV71-B5)**     QEKDLEYGAC PNNMMGTFSV RTVGSSKSKY PLVIRIYMRM KHVRAWIPRP MRNQNYLFKA
SiICRC05/TH/2014(EV71-B5)       .......... .......... .......... .......... .......... ..........
SiICRC07/TH/2014(EV71-B5)       .......... .......... .......... .......... .......... ..........
SiICRC01/TH/2014(EV71-C4a)**    .......... .......... ....T..... ...V...... .......... ..........
TH/MU-1/2015(EV71-C4a)          .......... .......... .......... .......... .......... ..........

                                     850        860
                                ----|----| ----|----| --
SiICRC10/TH/2011(EV71-B5)**     NPNYAGNSIK PTGASRAAIT TL
SiICRC05/TH/2014(EV71-B5)       .......... ......T... ..
SiICRC07/TH/2014(EV71-B5)       .......... ......T... ..
SiICRC01/TH/2014(EV71-C4a)**    .......... ......T... ..
TH/MU-1/2015(EV71-C4a)          .......... ......T... ..
```

Figure S3 (Continued). ** Test viruses for MN assay in this study
