# Supplementary material for: Seroprevalence of antibodies to enterovirus 71 and coxsackievirus A16 among people of various age groups in a northeast province of Thailand
Source: Virol J. 2018 Oct 16;15:158. doi: 10.1186/s12985-018-1074-8 (PMC6192276; doi:10.1186/s12985-018-1074-8)
Supplement: Supplementary file 5 — Figure S4. VP1-VP4 amino acid alignment of CA16. (PDF 250 kb) [file 12985_2018_1074_MOESM5_ESM.pdf]

```
                              VP4 (1 - 69)
                                      10         20         30         40         50         60
                              ....|....| ....|....| ....|....| ....|....| ....|....| ....|....|
SiICRC06/TH/2011(CA16-B1a)**  MGSQVSTQRS GSHENSNSAS EGSTINYTTI NYYKDAYAAS AGRQDMSQDP KKFTDPVMDV
TH/MUMT-1/2015(CA16-B1a)      .......... .......... .......... .......... .......... ..........
SiICRC01/TH/2012(CA16-B1b)**  .......... .......... .......... .......... .......... ..........
TH/MUMT-2/2015(CA16-B1b)      .......... .......... .......... .......... .......... ..L.......

                                         VP2 (70 - 323)
                                      70         80         90        100        110        120
                              ....|....| ....|....| ....|....| ....|....| ....|....| ....|....|
SiICRC06/TH/2011(CA16-B1a)**  IHEMAPPLKS PSAEACGYSD RVAQLTIGNS TITTQEAANI VIAYGEWPEY CPDTDATAVD
TH/MUMT-1/2015(CA16-B1a)      .......... .......... .......... .......... .......... ..........
SiICRC01/TH/2012(CA16-B1b)**  .......... .......... .......... .......... .......... ..........
TH/MUMT-2/2015(CA16-B1b)      .......... .......... .......... .......... .......... ..........

                                     130        140        150        160        170        180
                              ....|....| ....|....| ....|....| ....|....| ....|....| ....|....|
SiICRC06/TH/2011(CA16-B1a)**  KPTRPDVSVN RFFTLDTKSW AKDSKGWYWK FPDVLTEVGV FGQNAQFHYL YRSGFCVHVQ
TH/MUMT-1/2015(CA16-B1a)      .......... .......... .......... .......... .......... ..........
SiICRC01/TH/2012(CA16-B1b)**  .......... .......... .......... .......... .......... ..........
TH/MUMT-2/2015(CA16-B1b)      .......... .......... .......... .......... .......... ..........

                                     190        200        210        220        230        240
                              ....|....| ....|....| ....|....| ....|....| ....|....| ....|....|
SiICRC06/TH/2011(CA16-B1a)**  CNASKFHQGA LLVAVLPEYV LGTIAGGTGN ENSHPPYATT QPGQVGAVLT HPYVLDAGIP
TH/MUMT-1/2015(CA16-B1a)      .......... .......... .......... .......... .......... ..........
SiICRC01/TH/2012(CA16-B1b)**  .......... .......... .......... .......... .......... ..........
TH/MUMT-2/2015(CA16-B1b)      .......... .......... .......... .......... .......... ..........

                                     250        260        270        280        290        300
                              ....|....| ....|....| ....|....| ....|....| ....|....| ....|....|
SiICRC06/TH/2011(CA16-B1a)**  LSQLTVCPHQ WINLRTNNCA TIIVPYMNTV PFDSALNHCN FGLLVIPVVP LDFNTGATSE
TH/MUMT-1/2015(CA16-B1a)      .......... .......... .......... .......... .....V.... ..........
SiICRC01/TH/2012(CA16-B1b)**  .......... .......... .......... .......... .......... ..........
TH/MUMT-2/2015(CA16-B1b)      .......... .......... .......... .......... .......... ..........

                                                    VP3 (324 - 565)
                                     310        320        330        340        350        360
                              ....|....| ....|....| ....|....| ....|....| ....|....| ....|....|
SiICRC06/TH/2011(CA16-B1a)**  IPITVTIAPM CAEFAGLRQA VKQGIPTELK PGTNQFLTTD DGVSAPILPG FHPTPPIHIP
TH/MUMT-1/2015(CA16-B1a)      .......... .......... .......... .......... .......... ..........
SiICRC01/TH/2012(CA16-B1b)**  .......... .......... .......... .......... .......... ..........
TH/MUMT-2/2015(CA16-B1b)      .......... .......... .......... .......... .......... ..........

                                     370        380        390        400        410        420
                              ....|....| ....|....| ....|....| ....|....| ....|....| ....|....|
SiICRC06/TH/2011(CA16-B1a)**  GEVHNLLEIC RVETILEVNN LKTNETTPMQ RLCFPVSVQS KTGELCAAFR ADPGRDGPWQ
TH/MUMT-1/2015(CA16-B1a)      .......... .......... .......... .......... .......... ..........
SiICRC01/TH/2012(CA16-B1b)**  .......... .......... .......... .......... .......... ..........
TH/MUMT-2/2015(CA16-B1b)      .......... .......... .......... .......... .......... ..........

                                     430        440        450        460        470        480
                              ....|....| ....|....| ....|....| ....|....| ....|....| ....|....|
SiICRC06/TH/2011(CA16-B1a)**  STILGQLCRY YTQWSGSLEV TFMFAGSFMA TGKMLIAYTP PGGNVPADRI TAMLGTHVIW
TH/MUMT-1/2015(CA16-B1a)      .......... .......... .......... .......... .......... ..........
SiICRC01/TH/2012(CA16-B1b)**  .......... .......... .......... .......... .......... ..........
TH/MUMT-2/2015(CA16-B1b)      .......... .......... .......... .......... .......... ..........

                                     490        500        510        520        530        540
                              ....|....| ....|....| ....|....| ....|....| ....|....| ....|....|
SiICRC06/TH/2011(CA16-B1a)**  DFGLQSSVTL VVPWISNTHY RAHARAGYFD YYTTGIITIW YQTNYVVPIG APTTAYIVAL
TH/MUMT-1/2015(CA16-B1a)      .......... .......... .......... .......... .......... ..........
SiICRC01/TH/2012(CA16-B1b)**  .......... .......... .......... .......... .......... ..........
TH/MUMT-2/2015(CA16-B1b)      .......... .......... .......... .......... .......... ..........
```

Figure S4 VP1-VP4 amino acid alignment of CA16. GenBank accession numbers: KX372335 for SiICRC06/TH/2011(CA16-B1a), MH879030 for TH/MUMT-1/2015(CA16-B1a), KX372336 for SiICRC01/TH/2012(CA16-B1b), and MH879031 for TH/MUMT-2/2015(CA16-B1b). ** Test viruses for MN assay in this study

```
                                                                   VP1 (566 - 862)
                                       550        560        570        580        590        600
                                ....|....| ....|....| ....|....| ....|....| ....|....| ....|....|
SiICRC06/TH/2011(CA16-B1a)**    AAAQDNFTMK LCKDTEDIEQ TANIQGDPIA DMIDQTVNNQ VNRSLTALQV LPTAANTEAS
TH/MUMT-1/2015(CA16-B1a)        .......... .......... .......... .......... .......... ..........
SiICRC01/TH/2012(CA16-B1b)**    .......... .......... .......... .......... .......... ..........
TH/MUMT-2/2015(CA16-B1b)        .......... .......... .......... .......... .......M.. ..........

                                       610        620        630        640        650        660
                                ....|....| ....|....| ....|....| ....|....| ....|....| ....|....|
SiICRC06/TH/2011(CA16-B1a)**    SHRLGTGVVP ALQAAETGAS SNASDKNLIE TRCVLNHHST QETAIGNFFS RAGLVSIITM
TH/MUMT-1/2015(CA16-B1a)        .......... .......... .......... .......... .......... ..........
SiICRC01/TH/2012(CA16-B1b)**    .......... .......... .......... .......... .......... ..........
TH/MUMT-2/2015(CA16-B1b)        .......... .......... .......... .......... .......... ..........

                                       670        680        690        700        710        720
                                ....|....| ....|....| ....|....| ....|....| ....|....| ....|....|
SiICRC06/TH/2011(CA16-B1a)**    PTTGTQNTDG YVNWDIDLMG YAQLRRKCEL FTYMRFDAEF TFVVAKPNGE LVPQLLQYMY
TH/MUMT-1/2015(CA16-B1a)        .......... .......... .......... .......... .......... ..........
SiICRC01/TH/2012(CA16-B1b)**    .......... .......... .......... .......... .......... ..........
TH/MUMT-2/2015(CA16-B1b)        .......... .......... .......... .......... .......... ..........

                                       730        740        750        760        770        780
                                ....|....| ....|....| ....|....| ....|....| ....|....| ....|....|
SiICRC06/TH/2011(CA16-B1a)**    VPPGAPKPTS RDSFAWQTAT NPSVFVKMTD PPAQVSVPFM SPASAYQWFY DGYPTFGEHL
TH/MUMT-1/2015(CA16-B1a)        ........K. .......... .......... .......... .......... ..........
SiICRC01/TH/2012(CA16-B1b)**    .......... .......... .......... .......... .......... ..........
TH/MUMT-2/2015(CA16-B1b)        .......... .......... .......... .......... .......... ..........

                                       790        800        810        820        830        840
                                ....|....| ....|....| ....|....| ....|....| ....|....| ....|....|
SiICRC06/TH/2011(CA16-B1a)**    QANDLDYGQC PNNMMGTFSI RTVGTEKSPH SITLRVYMRI KHVRAWIPRP LRNQPYLFKT
TH/MUMT-1/2015(CA16-B1a)        .......... .......... .......... .....I.... .......... ..........
SiICRC01/TH/2012(CA16-B1b)**    .......... .......... .......... .......... .......... ..........
TH/MUMT-2/2015(CA16-B1b)        .......... .......... .......... .......... .......... ..........

                                       850        860
                                ....|....| ....|....| ..
SiICRC06/TH/2011(CA16-B1a)**    NPNYKGNDIK CTSTSRDKIT TL
TH/MUMT-1/2015(CA16-B1a)        .......... .......... ..
SiICRC01/TH/2012(CA16-B1b)**    .......... .......... ..
TH/MUMT-2/2015(CA16-B1b)        .......... .......... ..
```

Figure S4 (Continued). ** Test viruses for MN assay in this study
